# Raman and near Infrared Spectroscopy for Quantification of Fatty Acids in Muscle Tissue—A Salmon Case Study

**DOI:** 10.3390/foods11070962

**Published:** 2022-03-26

**Authors:** Nils Kristian Afseth, Katinka Dankel, Petter Vejle Andersen, Gareth Frank Difford, Siri Storteig Horn, Anna Sonesson, Borghild Hillestad, Jens Petter Wold, Erik Tengstrand

**Affiliations:** 1 Nofima AS—The Norwegian Institute of Food, Fisheries and Aquaculture Research, Osloveien 1, NO-1431 Ås, Norway; katinka.dankel@nofima.no (K.D.); petter.andersen@nofima.no (P.V.A.); gareth.difford@nofima.no (G.F.D.); siri.storteig.horn@nofima.no (S.S.H.); anna.sonesson@nofima.no (A.S.); jens.petter.wold@nofima.no (J.P.W.); erik.tengstrand@nofima.no (E.T.); 2 Benchmark Genetics Norway, Bradbenken 1, NO-5003 Bergen, Norway; borghild@vikingaqua.no

**Keywords:** Raman spectroscopy, NIR spectroscopy, fatty acid composition, EPA, DHA, salmon, cage of covariance

## Abstract

The aim of the present study was to critically evaluate the potential of using NIR and Raman spectroscopy for prediction of fatty acid features and single fatty acids in salmon muscle. The study was based on 618 homogenized salmon muscle samples acquired from Atlantic salmon representing a one year-class nucleus, fed the same high fish oil feed. NIR and Raman spectra were used to make regression models for fatty acid features and single fatty acids measured by gas chromatography. The predictive performance of both NIR and Raman was good for most fatty acids, with R^2^ above 0.6. Overall, Raman performed marginally better than NIR, and since the Raman models generally required fewer components than respective NIR models to reach high and optimal performance, Raman is likely more robust for measuring fatty acids compared to NIR. The fatty acids of the salmon samples co-varied to a large extent, a feature that was exacerbated by the overlapping peaks in NIR and Raman spectra. Thus, the fatty acid related variation of the spectroscopic data of the present study can be explained by only a few independent principal components. For the Raman spectra, this variation was dominated by functional groups originating from long-chain polyunsaturated FAs like EPA and DHA. By exploring the independent EPA and DHA Raman models, spectral signatures similar to the respective pure fatty acids could be seen. This proves the potential of Raman spectroscopy for single fatty acid prediction in muscle tissue.

## 1. Introduction

Fatty acids (FAs) are important biological and nutritional building blocks, playing critical roles in metabolism, as structural components in cells and as building blocks for other molecules (e.g., for hormones) in humans. In this respect, essential FAs such as linolenic acid (LA) and α-linolenic acid (ALA) are of special interest since they are required for normal growth but cannot be synthesized by the human body. Derivatives of essential FAs, such as the long-chain (LC) omega-3 FAs eicosapentaenoic acid (EPA) and docosahexaenoic acid (DHA), are known for having beneficial health effects, especially in preventing and attenuating inflammatory disorders [1]. Fatty fish, such as Atlantic salmon, are particularly rich in LC omega-3 FAs and are therefore major sources of these nutrients in the human diet. The FA composition of the fillet largely reflects dietary composition [2] but is also affected by fish genetics [3,4]. Fish oils rich in EPA and DHA intended for feed are limited resources that in recent years have been replaced with vegetable oils deficient in EPA and DHA, but higher in LA and ALA, which has resulted in declines of EPA and DHA content in farmed salmon [5]. Genetic selection for increased LC omega-3 FA conversion and deposition in salmon is an alternative approach for the aquaculture industry. However, analysis of EPA and DHA in salmon fillets with gold standard analytical methods (i.e., gas chromatography (GC)) proves too laborious and costly to enable largescale measurements for feed and genetic trials. Documentation of FA composition is therefore of importance both from a producer perspective (e.g., for documenting quality and price) and from a feeding and breeding perspective.

Raman and near infrared (NIRS) spectroscopy have been extensively studied and used for food and food component analysis. Whereas NIRS systems have been commercially available and used in food industries for decades, and low-cost NIRS systems are becoming increasingly available, Raman spectroscopy has not until recent years gained attention as a robust, valid and economically feasible alternative in food industry environments. Raman is a vibrational spectroscopy technique based on fundamental stretching and deformation modes, whereas NIRS is based on the measurement of overtones and combinations of vibrational modes. The spectral bands of NIRS are therefore generally broader compared to Raman, giving NIRS a lower chemical selectivity. For both techniques, however, lipid signals are readily available in the spectra. Thus, both techniques have been extensively studied for quantification of fatty acid features and single fatty acids in fats and oils [6,7] and lipid rich matrices such as adipose tissue [8,9,10].

An intriguing aspect of spectroscopic FA analysis is that FA fingerprints are apparent even in matrices where other components such as protein and water are present in high concentration (e.g., muscle samples). NIR spectra are more affected by protein and water bands than Raman, but still quantitative FA features can be obtained from spectra of complex samples [11]. Sampling possibilities now provided by both techniques (i.e., sample coverage and penetration depths), makes representative analysis of FA features directly in muscle samples feasible. Cecchinato et al. [12] showed that NIRS could be used to predict individual fatty acids in minced beef meat with good correlations for the major FAs present. Prieto et al. [13] used a similar approach for prediction of FAs in intact beef samples, obtaining satisfying results for several individual FAs. Brown et al. [14] obtained moderate to good calibrations for EPA and DHA contents of minced and intact salmon muscle using NIRS. In all these mentioned studies, FA composition was provided as absolute contents (i.e., in percentage of total sample contents). Using a hand-held Raman system, Fowler et al. [15] showed that Raman spectroscopy could be used to predict major FA groups directly in lamb meat with high correlations. For salmon, Afseth et al. [16] showed that Raman can be used to obtain a prediction of total unsaturation level of FAs directly in ground salmon muscle with moderate correlations. In that study, the iodine value was used since this FA feature provides an overall number for the total FA unsaturation of a lipid-containing sample. Recently, Lintvedt et al. [17] showed the potential for in-line Raman to predict the summed EPA and DHA content directly in homogenized salmon fillets. For the two latter Raman studies, FA composition was provided as relative contents (i.e., in percentage of total FA contents).

In a pure oil system, there will always be various degrees of covariances between different FAs. This can for instance be related to biological lipid metabolism (i.e., when one fatty acid increases, another one decreases), cultivation or feeding regimes. In a set of muscle samples, variation in fat-, water-, and protein contents will come as an additional factor to the variation of the FAs. In spectroscopic analysis, these levels of variation will have to be considered together. Covariance structures that are found in the design or in the chemical reference data of a calibration set, will be conserved in the calibration models, and when applying the models to future data sets, the predictions will be linearly dependent on each other. This means that FAs that co-vary in a calibration set can never be predicted independently from each other by the calibration models. This effect has been termed “cage of covariance”, and the effect will compromise calibration robustness, validity and interpretation [18]. An apparent example from muscle analysis is as follows: an increase of individual FAs causes an increase of the total fat contents. This may enable predictions of individual FAs through the indirect relationship with fat contents. Such a relationship was recently illustrated in the NIR analysis of homogenized salmon muscle [19]. The effect has also previously been illustrated in transmission Fourier-transform infrared analysis of proteins [18] and FAs [20] in milk. High covariance between single fatty acids and iodine values in pig fat has also been demonstrated [21]. This illustrates a few of the challenges that needs to be considered in the development of rapid spectroscopic techniques for FA analysis in muscle.

The increased focus on cage of covariance in spectroscopic analysis sheds light on the limitations of using Raman and NIR spectroscopy for single FA analysis in muscle samples. Currently, there is no consensus in the literature on what can be achieved, and in a range of studies, regression models with high correlations to single FAs can be found. However, the interpretation and thereby the critical evaluation of what is actually measured, is often lacking. Thus, the aim of the present study was to critically evaluate the potential of using NIR and Raman spectroscopy for prediction of FA features and single FAs in salmon muscle. The study was based on Raman and NIR analysis of 618 homogenized salmon muscle samples acquired from Atlantic salmon representing one year-class of the SalmoBreed nucleus, fed the same high fish oil feed. NIR and Raman spectra were used to make regression models for FA features measured by GC. To the author’s knowledge, this is the first time NIR and Raman spectroscopy are compared for prediction of FA features in muscle tissue.

## 2. Materials and Methods

### 2.1. Salmon Samples

The salmon samples in the current study were obtained from a genetic screening survey on genetic effects of FA composition in muscle of Atlantic salmon [3]. The screening represented one year-class of the SalmoBreed nucleus, kept by Benchmark Genetics Norway AS. The fish were transferred to net pens in the sea at a mean weight of 113.1 g and harvested at a mean slaughter weight of 3605 g. In total, 668 fish reared under the same conditions were included in the original study. The salmon were fed a commercial broodstock feed with a high fish oil content and were fasted 13 to 14 days prior to slaughter. Muscle samples from the Norwegian Quality Cut (NQC) of the fillet were collected at harvest from each fish, frozen and stored at −20 °C. Reference and spectroscopic analyses were performed on homogenized samples of the NQC. 

### 2.2. Reference Analysis

Reference analyses were performed as described in Horn et al. [3]. Total lipids were extracted from homogenized NQC muscle samples according to the method described by Folch et al. [22]. Using 1 mL of the chloroform–methanol phase, FA composition of the total lipids was analyzed according to the method described by Mason and Waller [23] employing gas chromatography (GC) with flame ionization detection. Individual fatty acid methyl esters were identified by reference to established standards. The proportional content of each FA was expressed as a percentage of the total amount of FA in the analyzed sample. Iodine value (IV) was calculated by Equation (1)

(1) $$IV=M_{w}\left(I_{2}\right)\sum_{i=1}^{n}\frac{\#DB_{\left(i\right)}*V_{FAMe\left(i\right)}}{M_{w}\left(FAMe\right)},$$

where *M_w_* is molecular weight, *I*_2_ is iodine, *FAMe*(*i*) is fatty acid number *i*, #DB is the number of double bonds and *V_FAMEe_*_(*i*)_ is percentage of fatty acid number *i*.

### 2.3. Spectroscopic Analysis

Spectroscopic analysis was performed as described in Difford et al. [24]. Diffuse reflectance near-infrared spectra of homogenized salmon samples were obtained using FOSS NIRSystems XDS Rapid ContentTM Analyzer (FOSS Analytical A/S, Hillerød, Denmark). NIR spectra were obtained in reflectance mode with 32 scans per spectrum. Homogenized samples were randomized and measured in triplicates, with the average spectrum used for further analysis. The internal ceramic standard was used as a reference. The spectral range was from 400 nm to 2500 nm in 0.5 nm increments. To linearize the data, the NIR spectra were transformed from reflectance (R) units into absorbance (A = log10(1/R)) and Standard Normal Variate (SNV) pre-processed. For subsequent analysis, the spectral range from 1150 nm to 2500 nm was used.

Raman spectra were collected using a Kaiser RamanRXN2™ Multi-channel Raman analyzer (Kaiser Optical Systems, Inc., Ann Arbor, MI, USA) with a spectral resolution of 5 cm^−1^. The spectrometer was equipped with a 785 nm laser and PhAT probe with a laser spot size diameter of 6 mm. The spectra were recorded with a laser power set to 400 mW in the range of 300–1890 cm^−1^ with 0.3 cm^−1^ intervals and an exposure time of 15 s × 4. All homogenized muscle samples were randomized, and three replicate measurements were obtained for each sample. The instrument was controlled using the iC Raman version X software (Mettler Toledo, Greifensee, Switzerland). For pre-processing, extended multiplicative signal correction (EMSC) with a sixth order polynomial extension was used [25]. In short, the spectra were trimmed into the range 500 cm^−1^ to 1800 cm^−1^, and the EMSC correction was performed on all replicate spectra. The mean spectrum of all the replicate spectra was subjected to polynomial baseline correction (fourth order) and used as a reference in the EMSC correction. The average Raman spectrum for each sample was subsequently calculated and used for further analysis. 

### 2.4. Data Analysis

When all data were merged and samples with missing data were discarded, a total of 618 samples were used in the subsequent data analysis. All data analysis (i.e., PCA and PLS regression) was performed with MATLAB R2020b (MathWorks, Natick, MA, USA). The regression models were made with PLS using PLS Toolbox v. 8.9.1 (Eigenvector Research, Manson, WA, USA), and the number of components and the performance were estimated by seven-fold venetian blind cross-validation. In the NIR dataset, the regions which had systematic coefficients and are known to have signal from FAs in the regression coefficients were selected as regions of interest. The models were retrained using only the regions of interest, and if the performance was better, the new models were kept. The new models turned out to be better for all models except total fat, C18-0 and C16-1. Similar variable selection was performed in the Raman dataset, but no models showed improvement from the variable selection. Instead, a bottom-up variable selection was used, which showed small improvements for all FAs. The variables were grouped in sets of 100, and each set was tested with cross-validation. The best set was kept, and all remaining sets were added separately, tested with cross-validation, and the best additional set was kept. This was repeated until the performance did not increase further.

## 3. Results and Discussion

### 3.1. Reference and Spectroscopy Data

GC reference data for all salmon samples are provided in Table 1. Here only the FAs with mean concentrations higher than 2.0%, measured as percentage of total FA contents, are provided. It is expected that FAs present at lower concentrations to a large part will not be visible in the respective Raman and NIR spectra. The most abundant FAs in each class were C16-0 (SFA), C18-1 (n-9) (MUFA) and C18-2 (n-6) (PUFA), together these three FAs contributed to approx. 50% of the total FA content. The most abundant FAs are expected to considerably contribute to the total signal in NIR and Raman spectra, thus potentially skewing any modelling attempt in their direction if any other FAs are highly correlated.

**Figure 1:**
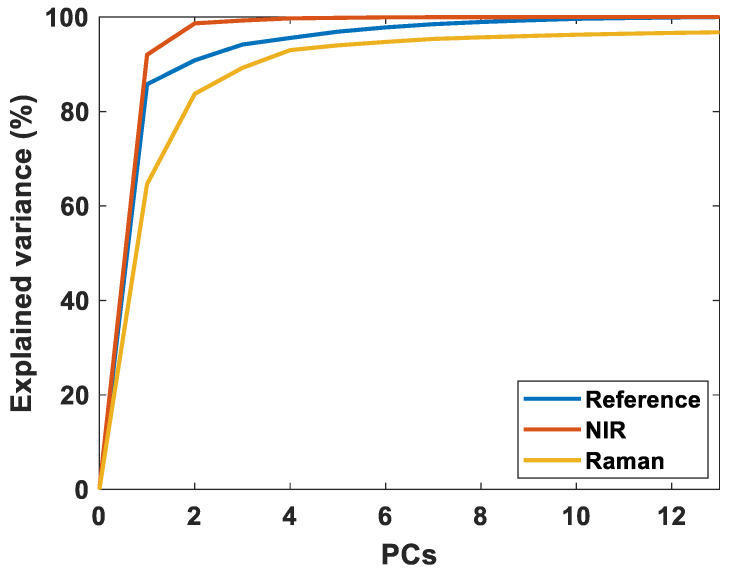
The explained variance of the reference data, NIR spectra, and Raman spectra, respectively. This figure only shows 13 components, because only 13 FAs are included in the data analysis.

Raw spectra of all NIR and Raman analysis in the study are provided in Supplementary Figure S1. Raman and NIR spectral bands related to fat contents and FA features have been extensively interpreted in the literature and will not be provided here. For the interested reader, comprehensive overviews can be found elsewhere [10,26]. A natural initial question is what type of information can be extracted from the respective spectra. Figure 1 shows the variance explained by PCA of the Raman spectra, the NIR spectra and the references, respectively. As seen in the figure, the variance explained by the first NIR components is substantially higher than for the references and Raman spectra. The reason for this is that much of the variance in the NIR spectra origins from strong signals from water and proteins, and from light scattering. Thus, for NIRS, a large part of the spectral information is connected to the bulk composition and is explained by a few components. In Raman, the chemical information is better resolved, and more components are required to explain the variance. Additionally, for Raman, signals from water and proteins are less pronounced in the spectra. Thus, in subsequent regression analysis, the Raman models are expected to use fewer components than NIRS since the chemical information is better resolved and the FA information is more dominant. It is also important to note that with only 5–6 components, most of the variance of the references are explained. This is a clear indication of the degree of independent FA variation in this data set.

### 3.2. Regression Results

**Figure 2:**
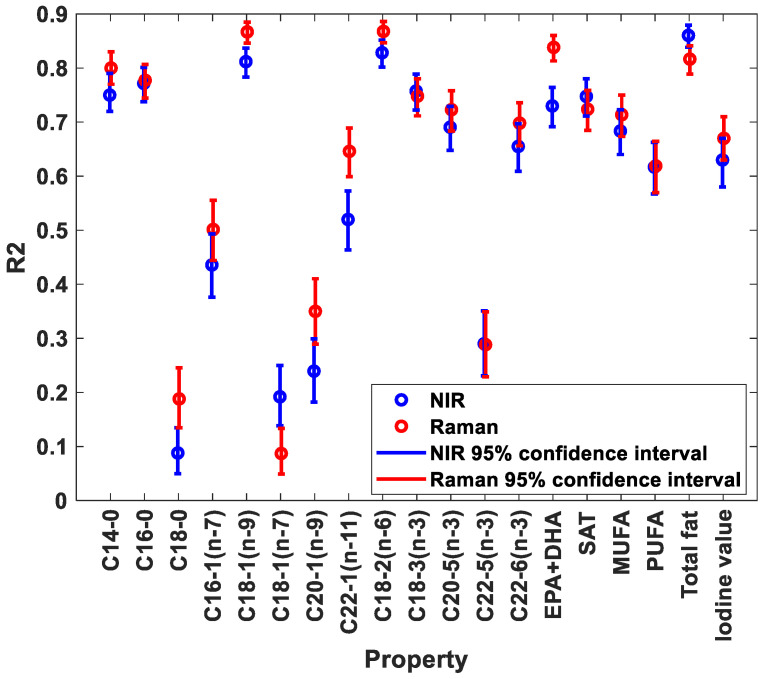
R^2^ values of predicted FAs as percentage of fat for NIR and Raman spectroscopy.

A comparison of predictive performance of NIRS and Raman for FA contents (as percentage of total FA contents) is provided in Figure 2. The complete overview of regression results is provided in Supplementary Tables S1 and S2. As seen from the figure, the predictive performance of both NIRS and Raman was good for most FAs, with R^2^ above 0.6. Among the saturated FAs, C14-0 and C16-0 showed best predictive performance. C18-1(n-9) was the only monounsaturated FA that provided good predictive performance. Among the polyunsaturated FAs, both C18-2(n-6), C18-3(n-3), EPA and DHA showed good predictive performance. FAs with poor predictive performances all had low variation in the reference values, with standard deviations at or below 0.3. The figure also shows that for most FAs, Raman performs better than NIRS. 

**Figure 3:**
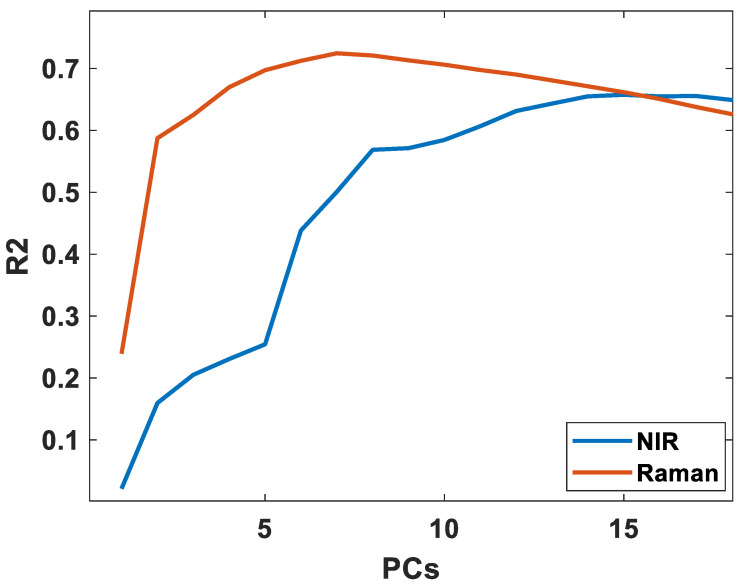
The cross-validated performance of DHA using NIR and Raman spectroscopy.

Another important aspect in predictive performance is model complexity (i.e., number of PLS factors used in the regression models). These results are also provided in Supplementary Tables S1 and S2. All NIR FA models used more components than the respective Raman models. Most NIR models had poor performance with less than 12 components, an average of 15 components was used, and all good FA models showed improvement up to 14 components. In contrast, most Raman models required seven to nine components, with an average of seven components during cross validation. To illustrate this, the cross-validated performance of the NIR and Raman models for DHA is provided in Figure 3. In Raman the predictive performance becomes high rapidly with the increasing inclusion of a comparatively smaller number of PLS components, then reaches an optimum, and subsequently starts dropping. In NIR, the performance increases until the 14th component, with a strong increase until the 12th. This further indicates that Raman has more dominant and more well-resolved information on FAs, potentially leading to more robust models, as also indicated in the discussion of Section 3.1.

### 3.3. Interpretation of Regression Models 

**Figure 4:**
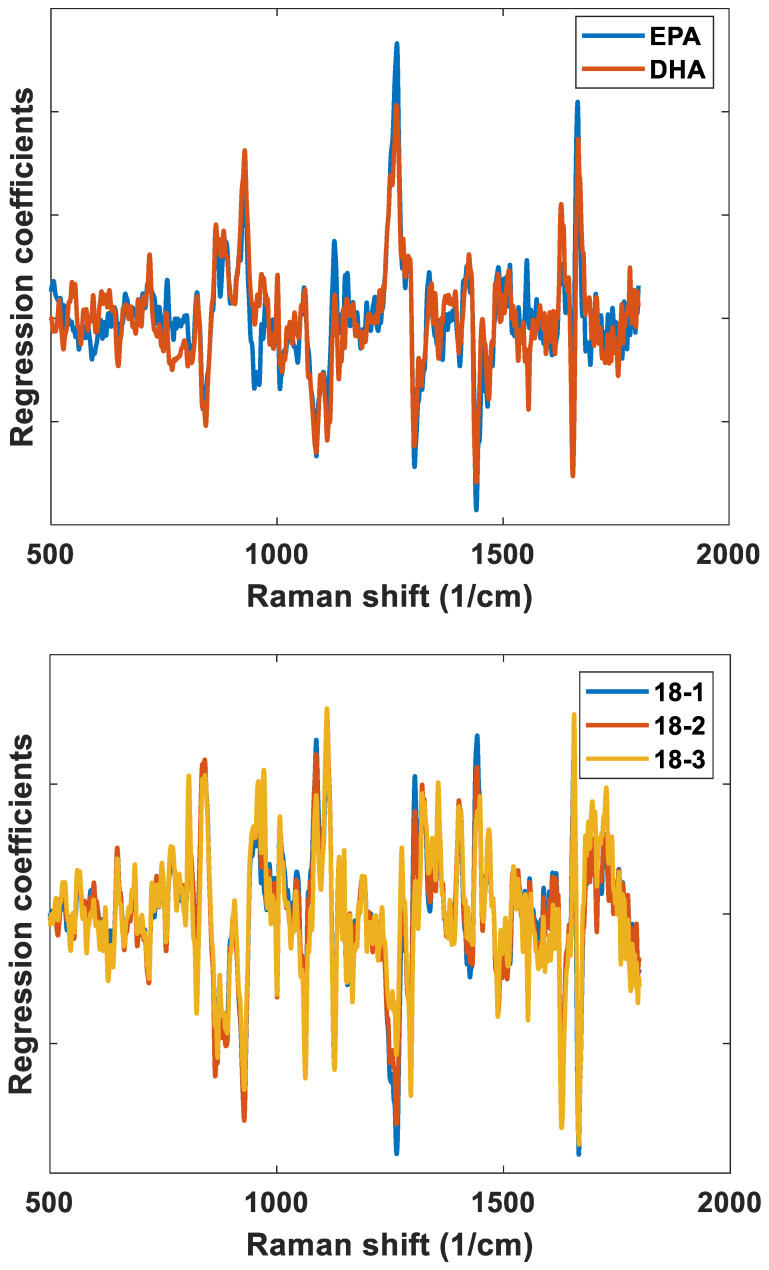
Regression coefficients obtained from Raman regression models of EPA and DHA (**upper plot)**, and 18:1(n-9), 18:2(n-6) and 18:3(n-3) (**lower plot**). All regression coefficients are obtained from regression models using five factors and have been normalized to simplify the comparison.

One of the most important aspects in spectroscopic measurements of minor components, such as FAs in muscle tissue, is unravelling the chemical logic behind the predictive performances obtained. In the subsequent discussion, two features will be used for this purpose: (1) the correlation coefficients of every pair of FAs in the sample set (as provided in Supplementary Figures S2 and S3); and (2) the regression coefficients of the regression model of each FA. Only the FAs providing the highest predictive performances according to Figure 2 have been considered. Of these FAs, EPA and DHA both have very high degree of unsaturation: five and six double bonds, respectively. The regression coefficients of their respective Raman regression models are provided in Figure 4a. First of all, the EPA and DHA models show the spectral features expected for polyunsaturated fatty acids: positive bands related to unsaturated functional groups around 1665 cm^−1^ (C = C stretch), 1266 cm^−1^ (symmetric = C-H rock), 935 cm^−1^ (alkene C-H deformation), and negative bands related to saturated functional groups around 1440 cm-1 (CH2 scissoring), 1300 cm^−1^ (CH2 twist), 1122 cm^−1^ (C-C stretch) and 1066 cm^−1^ (C-O stretch) [26]. EPA and DHA are indeed similar chemically, and in the present data set, the two FAs have a moderately high correlation of 0.51 (based on the reference data). Thus, from both of these viewpoints, the FAs will be difficult to separate by spectroscopy. However, the pure fatty acid Raman spectra of EPA and DHA are provided in Supplementary Figure S4, and here we also see minor differences between them, such as, for instance, a small shift in the C = C stretching vibration at 1665 cm^−1^ (moving from 1665 cm^−1^ for EPA to 1666 cm^−1^ for DHA) and a relatively higher intensity of the band at 935 cm^−1^ for DHA. When zooming into Figure 4a, these are the same changes that can be seen in the corresponding regression coefficients. This illustrates that even though the regression models are bound by the covariance structure of the reference data, there is still information available at the single FA level. 

For 18-1(n-9), 18-2(n-6) and 18-3(n-3), the regression coefficients are provided in Figure 4b. As seen in the figure, the coefficients are very similar. This is first of all related to the fact that all three FAs are highly correlated (i.e., 0.95 between 18-1(n-9) and 18-2(n-6), and 0.88 between 18-2(n-6), and 18-3(n-3)), as shown in Supplementary Figure S2. This explains why 18-3(n-3) is the only FA of the ones present with low concentration that shows high predictive performance according to Figure 2. Overall, the coefficients are also more difficult to interpret, and many of the bands explaining the EPA and DHA models are going in the opposite direction even though all fatty acids contain unsaturation, for instance, 1266 cm^−1^ (symmetric = C-H rock), which is negative in these models, and 1122 cm^−1^ (C-C stretch) and 1066 cm^−1^ (C-O stretch), which are positive. This may be related to the fact that there is a high negative correlation between EPA/DHA and 18-1(n-9) in the present dataset, as shown in Supplementary Figure S2. However, one intriguing aspect is the C = C stretching vibration, which here can be found around 1655 cm^−1^, i.e., at lower Raman shifts than for EPA and DHA. This is expected, and it is generally known that the frequency of the C = C stretch is very sensitive to changes in the level of unsaturation. The most abundant saturated fatty acid, namely C16-0, also reveals a large R^2^ value. It seems, however, that the regression coefficients are highly dependent on covariance with C18:1(n-9), exhibiting almost mirror images of each other’s regression coefficients (data not shown).

For the calculated FA features, i.e., SAT, MUFA, PUFA, iodine value and sum of EPA and DHA, the regression coefficients follow from the above discussion on interpretation of single FA features. The regression coefficients are provided in Supplementary Figure S5, and from the reference correlations provided in Supplementary Figure S2, it is clear that the sum of EPA and DHA resembles closely the EPA and DHA models. This also goes for the iodine value, even though the predictive performance is lower. Since PUFA is not dominated by EPA and DHA, the other polyunsaturated FAs are dominating the variation in PUFA. For MUFA, the regression coefficients resemble the regression coefficients of 18-1(n-9) since this is the dominating monounsaturated FA. In the same way, SAT is closely related to the contents of 14-0 and 16-0. 

For NIRS, the model interpretation is far more difficult than for Raman. This is related to the fact that Raman provides better resolved chemical information than NIRS and that far more components are used to model FAs using NIRS compared to Raman. Representative regression coefficients are provided in Supplementary Figure S6. What can be gained from these coefficients is that there is indeed specific variation of coefficients related to the different FA models. However, the origin of the bands and the minor variations seen is difficult to establish. 

### 3.4. FAs and Fat Contents

**Figure 5:**
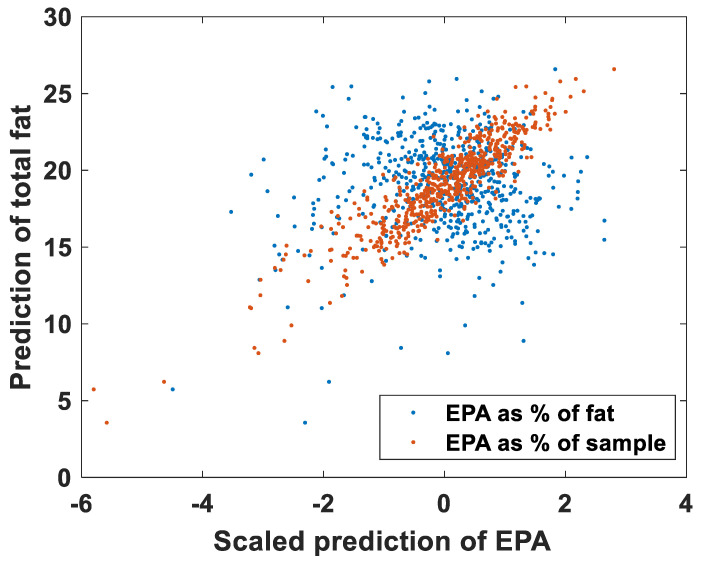

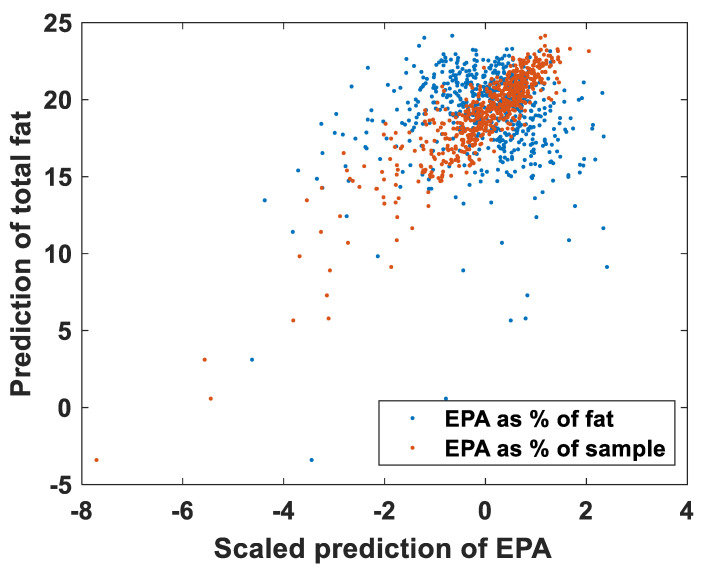
The covariance of EPA and fat predictions shown for NIR (**upper plot**) and Raman spectroscopy (**lower plot**) for EPA as percentage of fat and for EPA as percentage of sample.

FA contents can in principle be provided in two ways: (1) as proportional contents (i.e., in percentage of total FA contents); and (2) as absolute contents (i.e., in percentage of total sample contents). In the previous results, all results are based on the proportional contents of FAs. When modelling the FAs as absolute contents, all FAs have good predictive performance, with R^2^ above 0.6 and averaging 0.77 for Raman, and above 0.75 and averaging 0.81 for NIRS (data not shown). Good predictive performance was also provided for FAs at very low concentrations. Interestingly, all the FAs have correlations with fat contents above 0.57 when predicted as percentages of the sample, both for NIR and Raman (mean correlations with fat contents were 0.83 for NIR and 0.87 for Raman). This indicates that the performance seen is related to total fat contents: all FAs covary with the fat content, making all models that model FAs as a percentage of the sample fat instead of individual FA contents. This is illustrated in Figure 5 for EPA contents. EPA has a correlation of 0.8 with fat contents when predicted as absolute contents, and 0.0 when predicted as proportional contents. Considering that the models for fat contents have R^2^ values of 0.82 and 0.86 for Raman and NIR, respectively, this is a good indication that all FA models predict fat when the FAs are expressed as absolute contents. When predicting the FAs as proportional contents, the mean correlations between the FA and total fat were much lower: 0.08 and 0.12. This can be further illustrated by calculating the proportional contents from the prediction of the absolute contents. The predictions of the absolute contents of DHA with NIRS have an R^2^ value of 0.78. The predictions of the relative contents have an R^2^ value of 0.66. The predictions of the absolute concentration divided by the total fat have an R^2^ value of 0.38, while the predictions of the proportional contents multiplied by the total fat have an R^2^ value of 0.91. In other words, the predictive performance increases by using proportional content, even if the end goal is to use absolute content.

Similar results have been discussed in a recent paper on NIR analysis and cage of covariance [19]. For Raman, this has not been shown before, but it follows naturally from the potential of using Raman spectroscopy for fat contents measurements [24,27]. In the present study, NIR performs better than Raman spectroscopy when predicting FAs as absolute contents, and it is expected that this is related to the higher chemical selectivity of Raman, which is no longer relevant when the correlation with fat is so strong.

### 3.5. General Discussion

There are two special challenges in the spectroscopic analysis of FA composition: the covariance between FAs present, and their chemical similarity. Since many FAs covary with each other, data driven analysis approaches will mix up signals from the different FAs. In scenarios where new samples are expected to have different covariances this will be a problem e.g., when the goal is to alter individual FAs, such as in breeding. But for many operations new samples will have the same covariances, and this will pose no problem, e.g., quality inspection of existing lines. The problem with mixed up signals is exacerbated by the overlapping peaks in NIR and Raman spectroscopy. For instance, in GC analysis the different FAs have different retention times so the peak associated with each FA can be integrated and the covariance will not be a problem. The chemical similarity also poses a problem. Since the difference is only in the length of the carbon chains and the number and locations of the double bonds, all the FAs have similar spectra in both Raman and NIRS. The spectral similarities result in models that are more sensitive to disturbances. Despite these challenges, this study shows that NIR and Raman are both feasible tools for analysis of FAs in muscle tissue. In the present study both methods were measured on ground meat with bench top systems which require sample preparations and measurements time. However, rapid inline NIR systems are currently readily available and in use for grading and sorting Atlantic salmon fillets for quality parameters such as color and total fat contents. Lintvedt et al. [17] showed the potential for in-line Raman to predict the summed EPA and DHA content directly in homogenized salmon fillets. Here, the prediction performance reported for EPA + DHA (R^2^ = 0.91; RMSECV = 0.41) is higher than in the present study. In the Lintvedt study, the main variation in FA composition was related to different feeds, which can have contributed to the improved predictive performance. In another recent study on Rainbow trout (*Oncorhynchus Mykiss*), Raman micro-spectroscopy was used to predict EPA (R^2^ = 0.76) and DHA (R^2^ = 0.81) [9]. For this study, however, measurements were performed on adipose tissue and not in the muscle as such. 

Based on the results of the current study, Raman is likely more robust for measuring FAs compared to NIR. The Raman models require fewer components to reach high and optimal performance. The Raman models for single FAs also have generally higher performance. Of the nine FAs with an R^2^ of at least 0.4 for both NIR and Raman, three have higher performance with Raman with 99% confidence, and six with at least 95% confidence. Two of the models have a performance that is higher but with lower than 95% statistical confidence. Only one FA has a higher performance with NIR, and that is with less than 95% confidence. The NIR models also required more components to reach their optimal performance. Comparing the covariances between the FAs indicated that the higher number of components required by NIR was due to overlapping signals between covarying FAs. With a lower number of components for NIR, the fatty acids showed much higher covariances than between the references. With the selected number of components, NIR and Raman predictions have very similar covariances between the FAs, and only 8% higher covariances than the references. 

The current study reveals clearly how difficult it is to unravel which FAs could actually be measured using a spectroscopic tool. Clearly, from the near 30 FAs that are analyzed by standard GC measurements, only a few FAs and FA features can be individually predicted. These possibilities will rely on variation, concentration and not least, the functional groups of a given FA. Even though there are only subtle chemical differences between EPA and DHA, the Raman regression models of the current dataset indicate that individual predictions of their contents are possible. This is seen among other things related to the highly Raman active C = C stretching of these polyunsaturated fatty acids. This can also explain why EPA + DHA is the FA feature that is “best” predicted of all FA features calculated. Moreover, even though the concentration of 18-1(n-9) is two to three folds higher than EPA and DHA in the samples, and that there is a large negative correlation between contents of EPA + DHA and 18-1(n-9), the Raman models show that it is the unsaturation features of EPA and DHA that dominates the regression coefficients. This is natural considering the number of double bonds that is contained in EPA and DHA. However, since EPA and DHA have relatively low concentrations, calculated FA features such as MUFA and iodine value are not dominated by the variation in EPA and DHA. These features thus have lower predictive performances than DHA and EPA. This is contrary to the study of Berhe, where the FA variation in pigs were shown to be closely linked to the calculated iodine values when predicted with Raman spectroscopy [21]. This is most likely related to the fact that pigs have lower amounts of polyunsaturated FAs than salmon. 

The EPA and DHA contents remain important quality criteria for consumers due to their health benefits, but also for their health benefits for the fish themselves. At present no discrimination is made between fillets with high or low EPA and DHA content, but due to the limited contents of EPA and DHA in salmon, producers are looking into price differentiation systems based on the EPA and DHA contents. Hence, product documentation of these FAs will be of major importance. Since Atlantic salmon are one of the few aquaculture species with the ability to elongate and desaturate LA and ALA found in plant-based ingredients into EPA and DHA, and since EPA and DHA content are significantly heritable [3], there is interest in selective breeding for future generations of Atlantic salmon with enhanced EPA and DHA conversion capacity. However, a requirement of selective breeding is the need to accurately record EPA and DHA in large genetic cohorts of 10^3^–10^4^ relative individuals each year; this requires rapid and cost-effective solutions [28]. To this end both NIR and Raman have shown extreme potential for fat content [24]. More recently, a study in another salmonid, Rainbow trout (*Oncorhynchus Mykiss*), illustrated the potential of Raman micro-spectroscopy to predict EPA and DHA contents in a genetic cohort of 268 fish of which EPA was significantly heritable (h^2^ = 0.16 ± 0.05), indicating the genetic progress that can be made for this trait [29]. Future work is therefore needed to explore the feasibility of Raman and NIR prediction of FAs for breeding in Atlantic salmon.

## 4. Conclusions

This study evaluates the potential of using NIR and Raman spectroscopy for prediction of FA features and single FAs in salmon muscle. The predictive performance of both techniques was good for most FAs, with R^2^ above 0.6. Overall, Raman performed marginally better than NIR, and since the Raman models generally required fewer components than respective NIR models to reach high and optimal performance, Raman is likely more robust for measuring FAs compared to NIR. The study also shows that the FA related variation of the spectroscopic data can be explained by only a few independent principal components. For the Raman spectra, this variation is dominated by functional groups originating from long-chain polyunsaturated FAs such as EPA and DHA. By exploring the independent EPA and DHA Raman models, spectral signatures similar to the respective pure FAs could be seen. This proves the potential of Raman spectroscopy for single FA prediction in muscle tissue. 

## Figures and Tables

**Table 1:** Results from GC reference analysis of FAs and summed FA properties (percentage of total FA contents).

Property	Mean	Min	Max	Range	SD ^1^
C14-0	3.4	2.1	4.0	1.9	0.3
C16-0	11.7	8.7	12.9	4.2	0.6
C18-0	2.7	2.3	3.8	1.5	0.2
C16-1 (n-7)	4.0	2.3	4.9	2.6	0.3
C18-1 (n-9)	30.5	26.3	39.5	13.2	1.8
C18-1 (n-7)	3.0	1.8	4.3	2.5	0.3
C20-1 (n-9)	3.7	2.8	4.6	1.8	0.3
C22-1 (n-11)	3.1	1.3	4.5	3.2	0.5
C18-2 (n-6)	9.6	8.1	13.4	5.3	0.7
C18-3 (n-3) (ALA)	3.4	2.9	4.4	1.5	0.2
C20-5 (n-3) (EPA)	5.1	2.6	6.5	3.9	0.5
C22-5 (n-3)	2.3	1.6	2.8	1.2	0.2
C22-6 (n-3) (DHA)	6.8	4.3	9.0	4.7	0.5
Sum of EPA and DHA	11.9	7.1	15.0	7.9	0.9
SFA ^2^	18.8	13.9	21.0	7.1	1.0
MUFA ^3^	49.0	44.8	54.7	9.9	0.9
PUFA ^4^	30.9	28.3	33.4	5.1	0.7
Iodine value	135.1	125.8	144.5	18.8	2.5

^1^ SD = standard deviation. ^2^ SFA = saturated fatty acids. ^3^ MUFA = monounsaturated fatty acids. ^4^ PUFA = polyunsaturated fatty acids.

## Data Availability

The data of the current study are available on request.

## Supplementary Materials

The following supporting information can be downloaded at: https://www.mdpi.com/article/10.3390/foods11070962/s1, Figure S1: Raw NIR and Raman spectra of all samples; Figure S2: Pairwise correlation between reference and predicted FA contents using Raman spectroscopy; Figure S3: Pairwise correlation between reference and predicted FA contents using NIRS; Figure S4: Raman spectra of pure EPA and DHA; Figure S5: Raman regression coefficients of summed FA features; Figure S6: NIRS regression coefficients of FA contents; Table S1: Raman PLS regression results; Table S2: NIRS PLS regression results.
