# Supplementary material for: Raman and near Infrared Spectroscopy for Quantification of Fatty Acids in Muscle Tissue—A Salmon Case Study
Source: Foods. 2022 Mar 26;11(7):962. doi: 10.3390/foods11070962 (PMC8997921; doi:10.3390/foods11070962)
Supplement: Supplementary file 1 [file foods-11-00962-s001.zip › foods-1629291-supplementary.pdf]

foods

Article

Raman and near infrared spectroscopy for quantification of fatty acids in muscle tissue – a salmon case study

Nils Kristian Afseth, Katinka Dankel, Petter Vejle Andersen, Gareth Difford, Siri Storteig Horn, Anna Sonesson, Borghild Hillestad, Jens Petter Wold and Erik Tengstrand

SUPPLEMENTARY MATERIALS

Figure S1. Raw spectra of all 618 samples obtained using NIR (upper plot) and Raman (lower plot) spectroscopy.

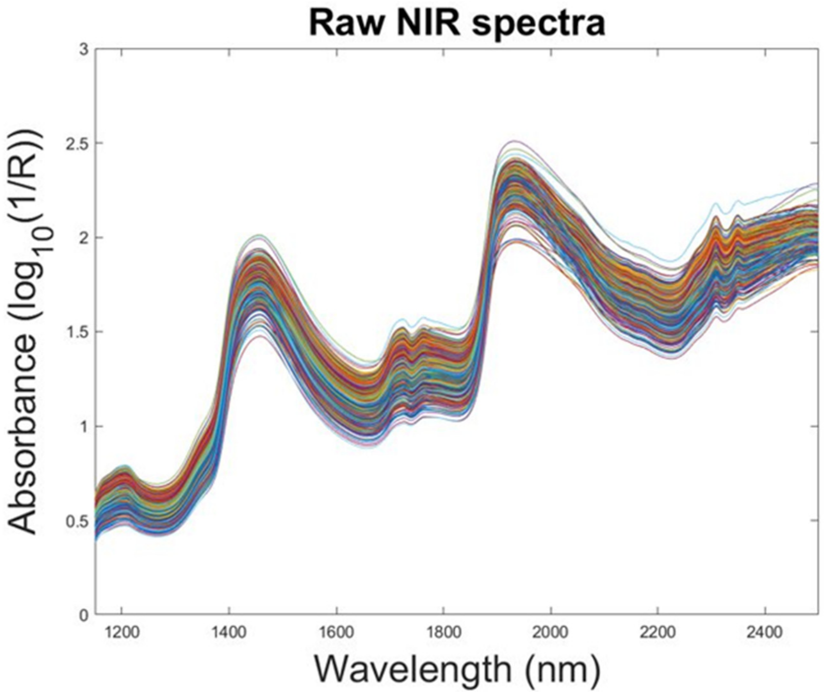

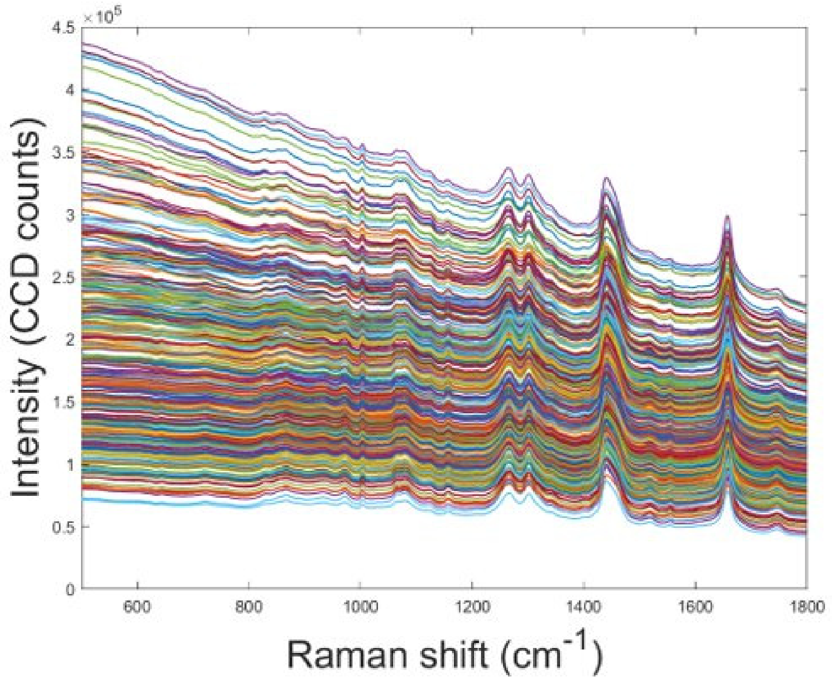

Figure S2. Pairwise correlation between individual FAs and PLS models for FA content based on Raman spectroscopy. The plot shows pairwise covariances between the FA predictions and references. The diagonal is the covariance between the prediction and the reference for a given FA. The elements above the diagonal show the covariances between the references of a pair of FAs. The elements below the diagonal show the covariances between the predictions of a pair of FAs.

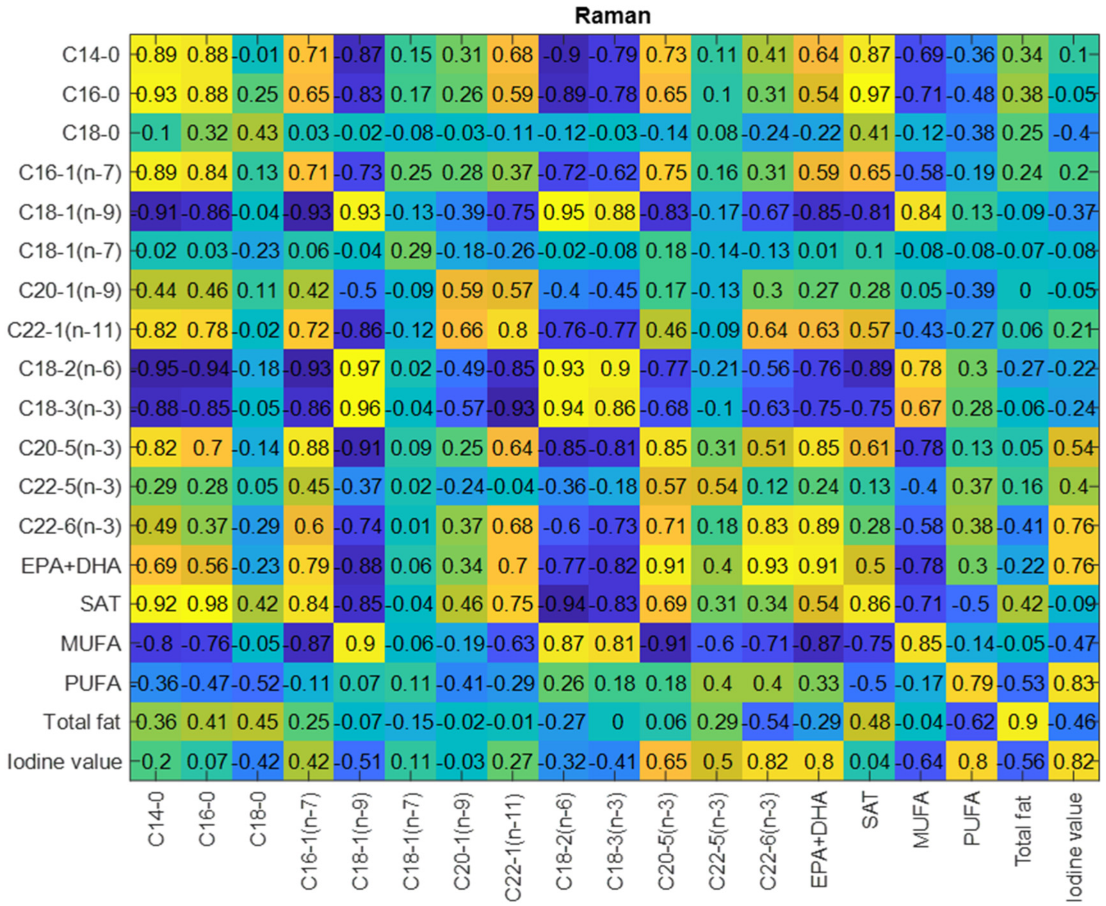

Figure S3. Pairwise correlation between individual FAs and PLS models for FA content based on NIR spectroscopy. The plot shows pairwise covariances between the FA predictions and references. The diagonal is the covariance between the prediction and the reference for a given FA. The elements above the diagonal show the covariances between the references of a pair of FAs. The elements below the diagonal show the covariances between the predictions of a pair of FAs.

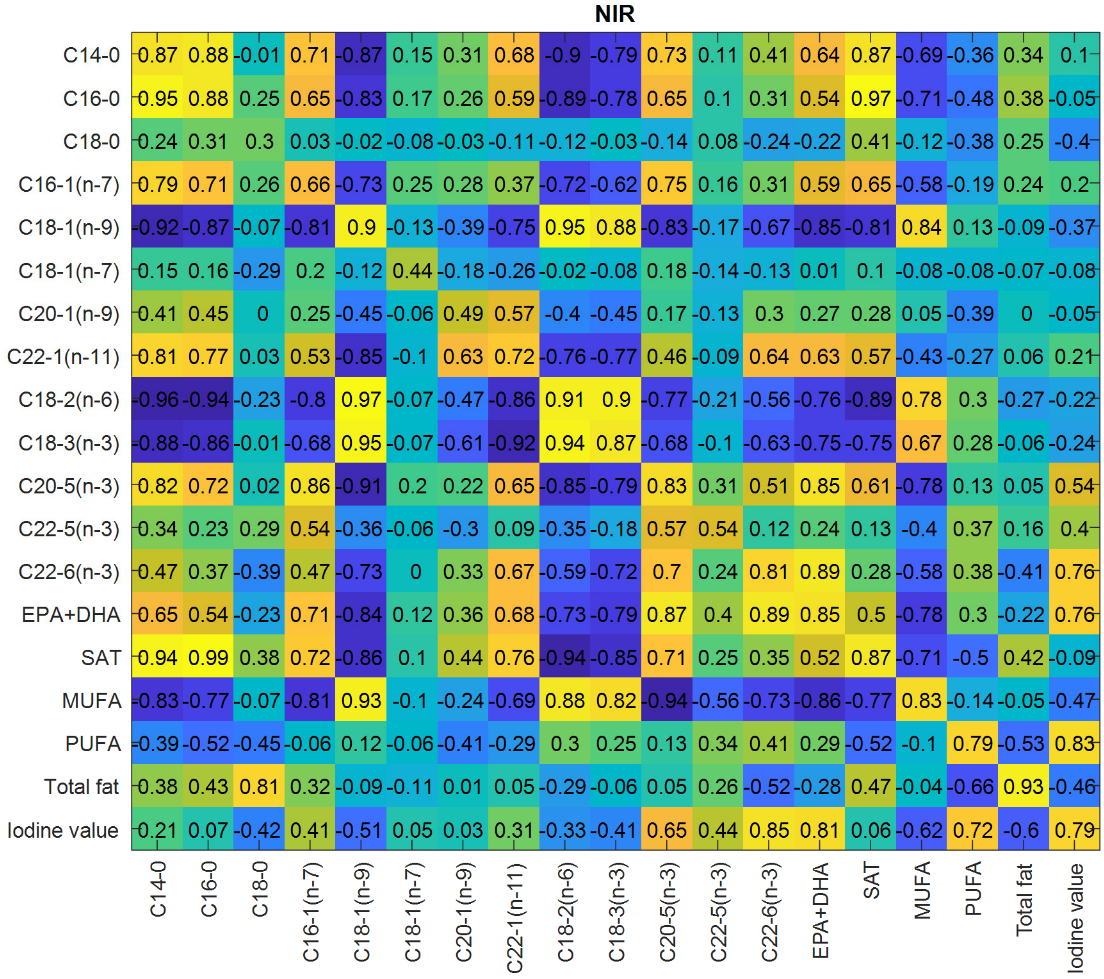

Figure S4. Raman spectra of pure EPA and DHA shown in blue and orange, respectively (upper plot). A magnification of the 1660 cm^{-1} peak in the Raman spectra are provided in the lower plot.

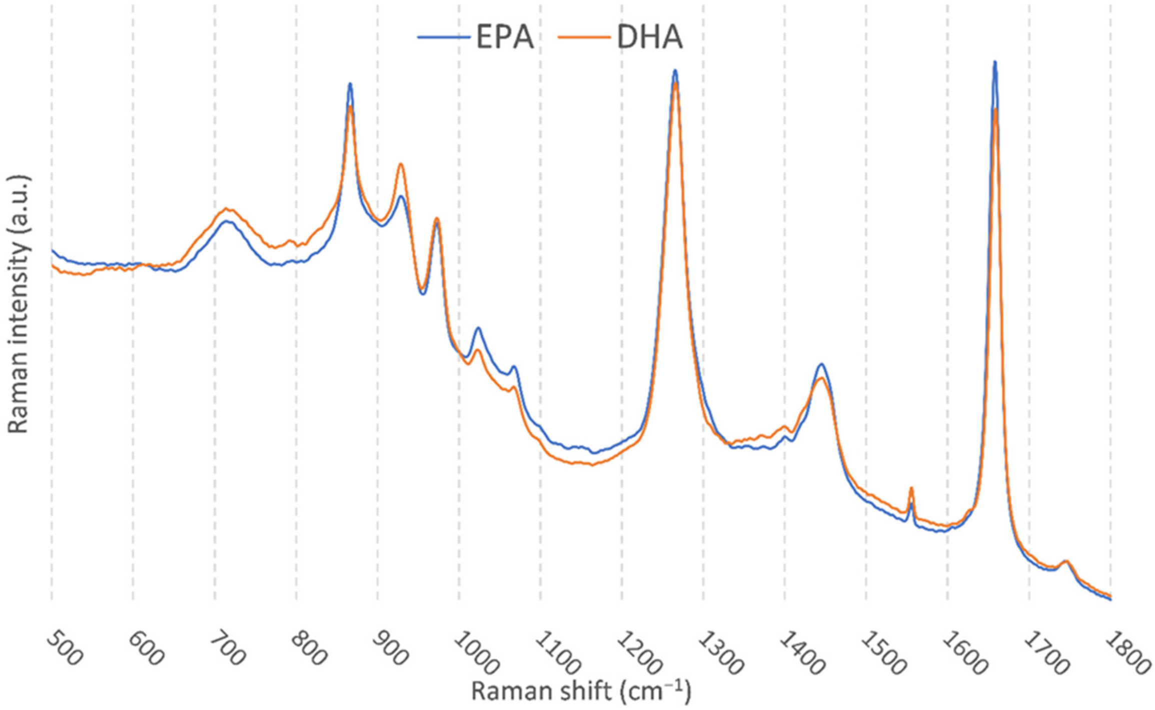

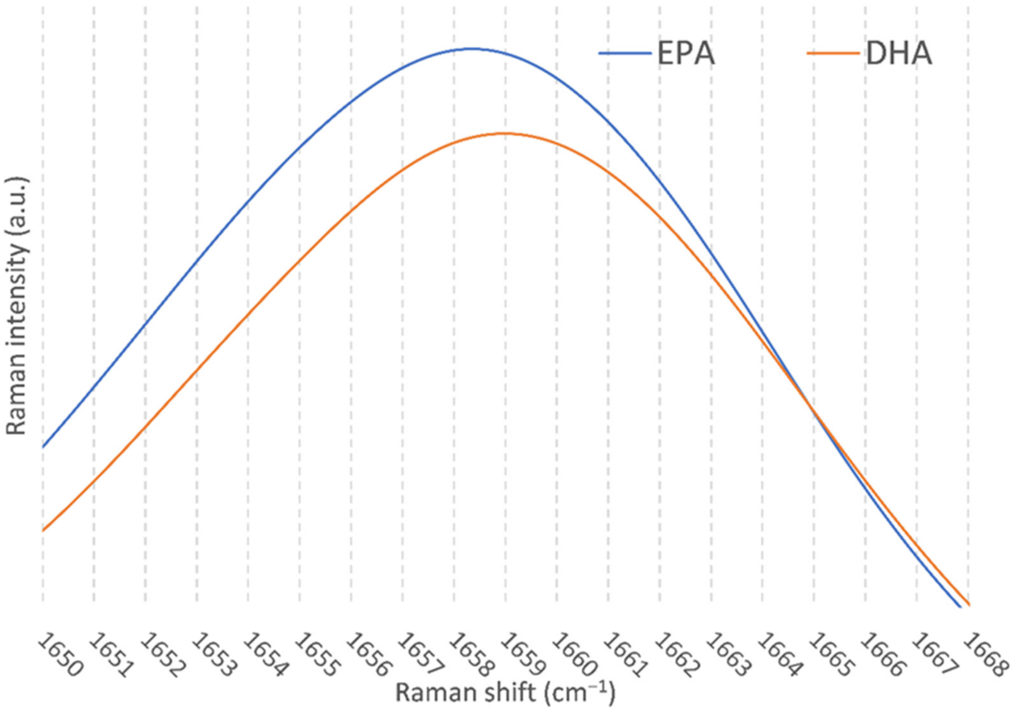

Figure S5. Regression coefficicents of summed fatty acid features obtained from Raman spectra using 5 component PLS models.

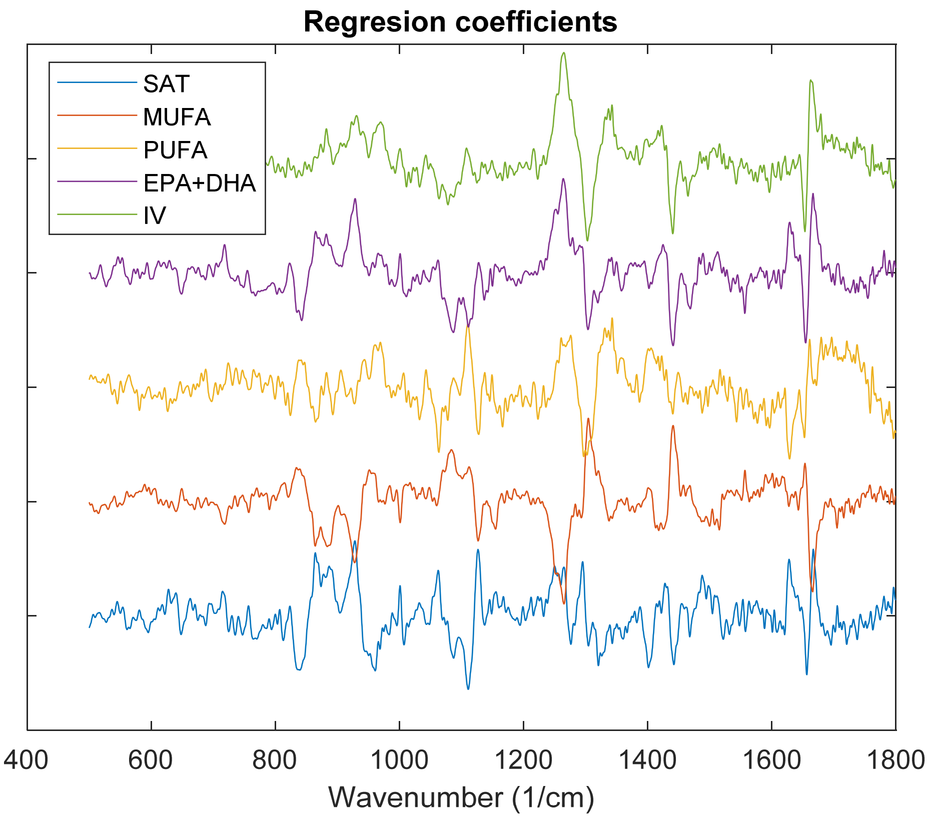

Figure S6. Regression coefficicents of summed fatty acid features, DHA and NIR obtained from NIR spectra.

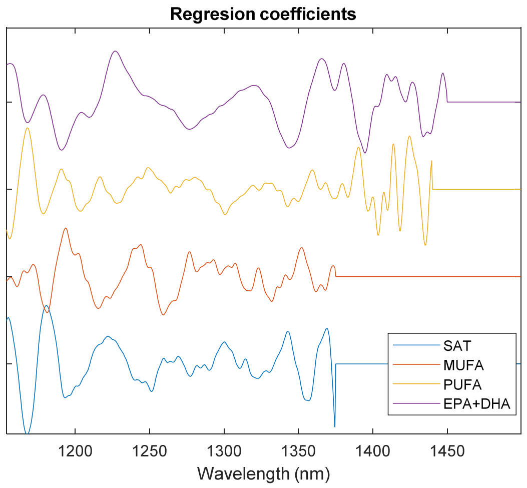

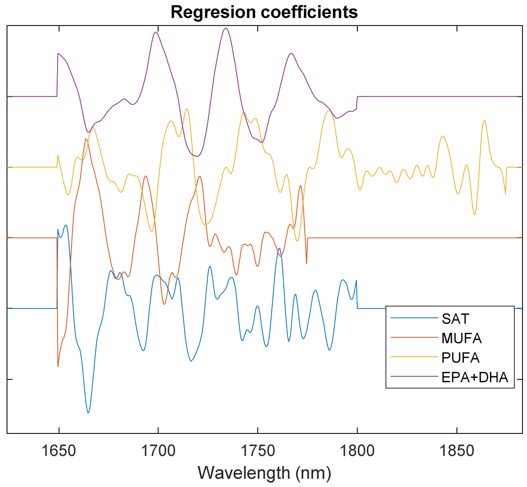

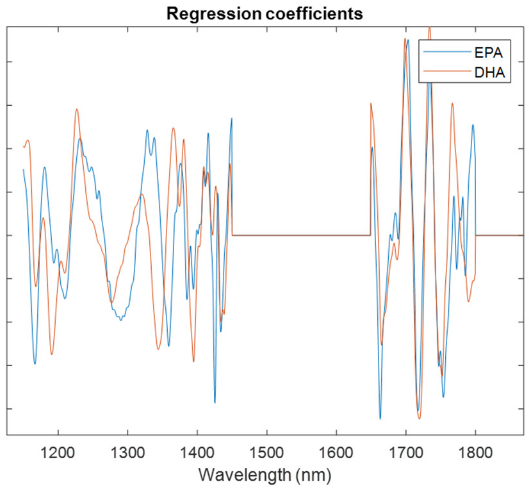

Table S1. PLS regression statistics for Raman models when predicting FA as percentage of total FA contents.

Name	PCs	R^2	95% lower bound	95% upper bound	SEP	95% lower bound	95% upper bound
C14-0	8	0.80	0.77	0.83	0.12	0.11	0.12
C16-0	9	0.79	0.76	0.82	0.29	0.28	0.31
C18-0	7	0.21	0.16	0.27	0.19	0.18	0.20
C16-1(n-7)	8	0.54	0.49	0.59	0.21	0.20	0.22
C18-1(n-9)	10	0.88	0.86	0.89	0.64	0.61	0.68
C18-1(n-7)	10	0.11	0.07	0.17	0.29	0.28	0.31
C20-1(n-9)	10	0.40	0.34	0.46	0.22	0.21	0.23
C22-1(n-11)	10	0.67	0.63	0.71	0.29	0.28	0.31
C18-2(n-6)	9	0.88	0.86	0.89	0.26	0.24	0.27
C18-3(n-3)	10	0.77	0.73	0.80	0.11	0.10	0.12
C20-5(n-3)	8	0.76	0.73	0.79	0.23	0.21	0.24
C22-5(n-3)	7	0.36	0.30	0.42	0.12	0.12	0.13
C22-6(n-3)	7	0.72	0.69	0.76	0.27	0.26	0.29
EPA+DHA	9	0.87	0.85	0.89	0.31	0.30	0.33
SAT	9	0.74	0.71	0.78	0.50	0.47	0.53
MUFA	9	0.73	0.69	0.77	0.48	0.45	0.50
PUFA	10	0.62	0.57	0.66	0.44	0.42	0.47

Table S2. PLS regression statistics for NIR models when predicting FA as percentage of total FA contents.

Name	PCs	R^2	95% lower bound	95% upper bound	SEP	95% lower bound	95% upper bound
C14-0	14	0.75	0.72	0.79	0.13	0.12	0.14
C16-0	17	0.77	0.74	0.80	0.31	0.29	0.33
C18-0	6	0.09	0.05	0.13	0.20	0.19	0.21
C16-1(n-7)	14	0.44	0.38	0.49	0.23	0.22	0.25
C18-1(n-9)	16	0.81	0.78	0.84	0.80	0.75	0.84
C18-1(n-7)	15	0.19	0.14	0.25	0.27	0.26	0.29
C20-1(n-9)	17	0.24	0.18	0.30	0.24	0.23	0.26
C22-1(n-11)	19	0.52	0.46	0.57	0.36	0.34	0.38
C18-2(n-6)	18	0.83	0.80	0.85	0.30	0.29	0.32
C18-3(n-3)	18	0.76	0.72	0.79	0.11	0.11	0.12
C20-5(n-3)	20	0.65	0.60	0.69	0.27	0.26	0.29
C22-5(n-3)	14	0.29	0.23	0.35	0.13	0.12	0.14
C22-6(n-3)	15	0.65	0.60	0.69	0.31	0.30	0.33
EPA+DHA	12	0.63	0.58	0.67	0.56	0.53	0.59
SAT	17	0.75	0.71	0.78	0.50	0.47	0.53
MUFA	16	0.68	0.64	0.72	0.52	0.49	0.55
PUFA	22	0.62	0.57	0.66	0.44	0.42	0.47
